# Mental Health Technologies: Designing With Consumers

**DOI:** 10.2196/humanfactors.4336

**Published:** 2016-01-28

**Authors:** Simone Orlowski, Ben Matthews, Niranjan Bidargaddi, Gabrielle Jones, Sharon Lawn, Anthony Venning, Philippa Collin

**Affiliations:** ^1^Flinders Human Behaviour & Health Research UnitDepartment of PsychiatryFlinders UniversityBedford ParkAustralia; ^2^Young and Well Cooperative Research CentreAbbotsford, VictoriaAustralia; ^3^School of Information Technology & Electrical EngineeringUniversity of QueenslandBrisbaneAustralia; ^4^School of MedicineUniversity of AdelaideAdelaideAustralia; ^5^Institute for Culture and SocietyUniversity of Western SydneyPenrithAustralia

**Keywords:** design thinking, participatory design, mental health, technology

## Abstract

Despite growing interest in the promise of e-mental and well-being interventions, little supporting literature exists to guide their design and the evaluation of their effectiveness. Both participatory design (PD) and design thinking (DT) have emerged as approaches that hold significant potential for supporting design in this space. Each approach is difficult to definitively circumscribe, and as such has been enacted as a process, a mind-set, specific practices/techniques, or a combination thereof. At its core, however, PD is a design research tradition that emphasizes egalitarian partnerships with end users. In contrast, DT is in the process of becoming a management concept tied to innovation with strong roots in business and education. From a health researcher viewpoint, while PD can be reduced to a number of replicable stages that involve particular methods, techniques, and outputs, projects often take vastly different forms and effective PD projects and practice have traditionally required technology-specific (eg, computer science) and domain-specific (eg, an application domain, such as patient support services) knowledge. In contrast, DT offers a practical off-the-shelf toolkit of approaches that at face value have more potential to have a quick impact and be successfully applied by novice practitioners (and those looking to include a more human-centered focus in their work). Via 2 case studies we explore the continuum of similarities and differences between PD and DT in order to provide an initial recommendation for what health researchers might reasonably expect from each in terms of process and outcome in the design of e-mental health interventions. We suggest that the sensibilities that DT shares with PD (ie, deep engagement and collaboration with end users and an inclusive and multidisciplinary practice) are precisely the aspects of DT that must be emphasized in any application to mental health provision and that any technology development process must prioritize empathy and understanding over innovation for the successful uptake of technology in this space.

## Introduction

In light of recent reports that there are almost as many mobile phone subscriptions (6.8 billion) as there are people on Earth (7 billion) [1], more humans are connected and have access to a wide range of information and services than ever before. In the context of this “increased access to information” the promise of the Internet and digital technologies is especially powerful in the prevention and treatment of mental health conditions, an area that has been historically impeded by issues of stigma and misinformation as well as disease-specific, geographical, and financial barriers to help-seeking and service engagement [2-5]. Despite growing interest in the promise of e-mental health preventive/treatment interventions, little supporting literature exists to guide their design and the evaluation of their effectiveness [6-8].

In line with an extensive literature on consumer participation in health care and mental health care more broadly [9-16], human-centered design processes have been identified as a method or set of techniques that assist with good design [17-22]. Both participatory design (PD) and design thinking (DT) have emerged as approaches that hold significant potential for supporting the design of technology-based youth e-mental health and well-being interventions [8,20,23-26]. For example, large-scale PD is embedded within Young and Well Cooperative Research Centre (CRC) [20,27] practice. The CRC combines end-user engagement and youth participation to “explore and understand the role of new and emerging technologies in the lives of young people” [28]. This paper provides a brief background of the evolution DT and PD, where differences in politics and agenda are explored. We then discuss the applicability of PD and DT to design of e-mental health interventions, particularly in the context of application by novice researcher/practitioners. Finally, we present 2 case studies and highlight similarities and differences in process and outcome, mind-set, and emphasis and draw learnings from each to inform design of e-mental health interventions.

### Participatory Design in Brief

PD practice has its earliest roots in Scandinavia where it was employed by computer scientists and systems designers initially in industrial workplaces to preserve the autonomy of employees facing significant changes to the organization of their work due to the introduction of new technologies. In this instance, improved outcomes were achieved due to the context-sensitive and future-oriented approach to the design of technological solutions developed by PD practitioners and the methods they used to involve workers in design [29-31]. A fundamental underpinning of Scandinavian PD was democratic participation in proposed changes to work and skill enhancement for workers [31]. One of the reasons PD gained international recognition was that a number of the early and archetypal examples of PD generated far-sighted and innovative solutions. (For example, the graphical user interface that was generated through the UTOPIA project in the early 1980s was clearly ahead of its time.) The methods of end user participation that were developed and shared out of these projects became adopted elsewhere as pathways to innovation—new means of designing successful and user-friendly systems. This gave rise to other more commercial (and less political) forms of PD, particularly in North America, where usability of software and products replaced the focus on workplace democracy [32].

In this Scandinavian context, the practice of PD was characterized by a 3-stage iterative design process aimed at unlocking a users’ tacit knowledge: (1) exploration of work; (2) discovery processes; and (3) prototyping. Each of these stages was organized and enacted with users [29]. More recently, variations of PD have been used in a range of contexts for a variety of purposes, with each implementation variously drawing on aspects of its practice (eg, applying PD as a general mind-set for design, or as a method, or adopting individual PD activities as design techniques [33]). PD, or “co-design” as it is called in its broad application, is now practiced within local communities, in companies and organizations, and between companies/organizations and their business partners and/or customers to tackle complex problems and promote innovation and user-centered design [33]. Increasingly PD has been employed in non-workplace contexts [34] by researchers without specific technical or design training as a means of improving the consumer experience in the design of new health interventions [20]. However, there is as yet little evidence as to whether these kinds of consumer participation in the design of new services succeed in improving the efficacy, implementation, and uptake of technology-based interventions [8].

### Design Thinking in Brief

Broadly speaking, DT is a term that refers to what designers and design researchers know about successful design processes (the first Design Thinking Research Symposium was held in 1991) [35-37]. In the past decade, however, it has become a term of reference for the mind-set, practices, and methods for generating innovative solutions, taking its starting point from ordinary people’s needs. Popularized by prominent design companies such as IDEO, DT has emerged as an articulation of a commercially successful human-centered design process. DT has been defined as “user-centered innovation with a focus on desirability” [38]. And, like PD, it emphasizes participation with and empathy toward users. Increasingly DT has influenced health care design, as well as delivery and training of the workforce [39-44].

DT reinforces the importance of multidisciplinary teams and their ability to generate a diversity of ideas. To harness the best ideas and output, team members are guided by an empathetic mind-set and methods, along with domain-specific knowledge. Naturally, this requires high levels of interpersonal communication. DT’s collaborative mind-set is underpinned by a bias toward action, which reinforces quick-and-dirty prototyping and a fail-early-and-often mentality [39,45]. DT is marketed for its ability to be successfully applied by novice practitioners using practical off-the-shelf toolkit [46,47]. DT is often associated with innovation as it attempts to uncover unidentified or unknown needs and offers a specific (and more prescriptive) way forward for the development of interventions that move beyond basic translation of paper-based processes and interventions onto a technology-based platform [33,45,48]. The Stanford d.School Bootcamp Bootleg is one of many available toolkits and is characterized by 5 design modes: empathize, define, ideate, prototype, and test [47]. Unsurprisingly, these modes neatly overlay the stages, or frameworks, proposed in traditional PD research [20,29]. The design-focused methods and mind-set, detailed in a resource such as the d.School Bootcamp Bootleg, provide an explicit and accessible method for health researchers to become exposed to a design mind-set and the possibility to innovate in circumstances that may be characterized as including incomplete or confusing information, which is often the starting point for intervention researchers.

### PD and DT in Health Care

If consumer involvement and/or a human-centered process is rightfully considered to be a part of good intervention design, then it is imperative to develop standards for and document cases of best practice. Hagen et al suggest a framework and techniques/methods for application of PD in a youth mental health intervention design context [20]. The guide articulates possible ways of integrating PD with more traditional evidence-based health research. The same adaptation work has not yet been done with respect to DT. Currently, the notion of applying a set of management processes developed in a commercial business and consulting context to sensitive fields such as youth mental health remains insufficiently interrogated with respect to benefit, risk, and applicability. For example, DT privileges in situ observation of end users to gain knowledge of subjective experience and insights for design. Privacy, confidentiality, and risk concerns make this type of brief observational engagement (by nonmental-health professionals) difficult to achieve in practice.

While the Hagen et al [20] PD framework is practical and accessible, it is unlikely that lay (nontechnical or nondesign) or inexperienced PD researchers would have the specific skill sets necessary to proficiently drive an iterative design process. This skill set in this area of research is particularly important when considering the predominantly consumerist rationale (ie, creating usable, effective, and efficient interventions) cited for employing participatory processes [8]. Sanders’ research has argued that the application of PD as a mind-set to guide predesign, discovery, and design initiatives “is best executed by very experienced research practitioners or by young, intuitive practitioners” [33]. This suggests that in the hands of lay and/or inexperienced researchers, PD may risk losing some of its power to create innovative solutions to future problems. This argument suggests a set of learnings and experiences that are tacit in the PD designer-researcher. It is worth emphasizing that while many of the staple PD methods (such as future workshops) appear easy enough to grasp, organize, and conduct, there is a great deal of skill that is required to successfully facilitate them. There is an important distinction between (1) the kinds of tools, processes, and methods used and (2) the mind-set underlying the approach taken. This raises questions around who is best placed to conduct the research and the kinds of interdisciplinary collaborations necessary for successful application of PD in health research contexts.

In contrast, the DT toolkits actively promote, and are arguably intended for, use by novice practitioners. For example, the method cards of a DT resource such as the Stanford d.School Bootcamp Bootleg [47] are deliberately specific in nature and are promoted in such way as to encourage wide dissemination and use. While this may be appealing for inexperienced researchers wishing to adapt design and innovation methods to e-mental health intervention design, it remains unknown just how effective they are in delivering on their promise of scaffolding novice practitioners through a successful design project. The lure of greater innovation in health care, as promised by the DT toolkits, is strong; the requisite skill and practice, however, involved in leading a DT project should not be underestimated, a point clearly highlighted in the following case study.

### Case Studies

Beyond the obvious differences in their respective agendas and politics, articulating universal or consistent distinctions between PD and DT practice can be difficult because their similarities are numerous. Both can be categorized under the umbrella term “human-centered design” and are linked to social innovation; collaborative, inclusive, and multidisciplinary practice; and iterative prototyping [31,45,49]. Moreover, DT and PD employ many of the same methods/techniques; for example, they both draw heavily from ethnographic fieldwork methods in their use of interviewing and observation and from design disciplines such as interaction design with techniques such as personas and scenarios [47,50]. Despite these macro similarities, subtle distinctions between the 2 do exist. These distinctions are best made obvious in their practical application; therefore, we present a case study of each to draw these out with the aim of better understanding their applicability to e-mental health and well-being intervention design.

The first case study describes a service design project carried out by an in-house design team at Kaiser Permanente, an American health care provider [51]. Kaiser Permanente is well known for its commitment to innovation and large-scale organizational application of DT [52]. The current case study describes use of DT in redesign of an initial DT service innovation—the Nurse Knowledge Exchange (NKE). This strategy aimed to improve nursing communication and handover (between shifts) in the organization’s hospitals. It did this by moving handovers at shift change from the employee breakroom to the patient bedside—a specific example of the type of innovation possible in application of DT. Five years later, the design team was tasked with the redesign of the NKE strategy due to incomplete and inconsistent uptake throughout the organization’s hospitals.

In their revision of NKE, Lin et al [51] describe a typical DT cycle—observing and interviewing followed by idea generation/design sessions, prototyping, and field testing. The process, as in most applications of DT, was rapid and expert-led (ie, controlled from start to finish by the design team), and it called on end users, which included staff from all organizational levels but no patients, for contributions at various stages—particularly during interviewing/observing and field testing. The end result was NKEplus.

The authors described heavy resistance to implementation of the NKEplus strategy outside the pilot site, which they attributed to skepticism in understanding exactly where the solutions that underpinned NKEplus originated. Lin and colleagues believed nurses throughout Kaiser Permanente’s hospitals did not see the need for change to their current handover practice and therefore had not bought into the NKEplus strategy. Lin et al [51] highlight that, in their organization, DT-based innovations and change are normally coupled with training support and formal changes to work roles and position descriptions. The rest of the case study details re-implementation of NKEplus, a process that resulted in higher uptake and buy-in for NKEplus organizationwide. This (ultimately more successful) re-implementation process shares a number of similarities with the PD case study, thus 2 case studies are described in parallel in the following section.

The second case study investigates adaptation of PD to a health context. Specifically, it concerns design of an eHealth portal to assist patients undergoing treatment for weight loss [53]. In contrast to the designer-led NKE redesign described above, the authors characterize the process as a design partnership with end users (which in this case were health care professionals and their patients). Moreover, as compared to the DT example, the PD design process took place in a research, not service, context that is typical of their respective applications.

As far as can be determined from the article, Das and Svanaes [53] began the project with a preconceived idea that an eHealth solution could assist patients undergoing weight loss treatment (similar to the DT example in which the overall aim was to improve nursing communication and handovers). Where the process differs from the DT example is that, as per the authors’ description, the actual design ideas came from the end users in future workshops that are typical of traditional PD practice. The health care professionals and patients who attended the future design workshops acknowledged the need for support in their treatment via self-help (eg, educational materials, reminders, asynchronous communication between provider and patient, etc) and suggested the possibility of an eHealth portal, which informed the prototypes that were presented to end users in subsequent workshops. The authors also investigated the differing priorities for various end users in the eventual design solution. Moreover, when an existing platform was presented to end users as a possible design solution, it was deemed insufficient and the researchers commissioned the custom build of a product that would meet end users’ requirements. This process took a year to complete, which amounts to a much longer timeframe compared to the rapid DT process described above.

In their second attempt to implement the NKEplus strategy, Lin et al [51] employed a more participatory version of DT via a “soft start” implementation process that made space for end user customization of the strategy. In contrast to initial implementation, the soft-start implementation was characterized by participation with “everyone on the same level conversing as peers” in the process. It also highlighted the fail-early-and-often mentality of DT, observable in the quick-and-dirty approach to trialing end-user-generated new ideas. Importantly, the authors ceded control over the solutions developed to the participants; for example, when participants raised concerns or criticisms with the proposed changes (or addressed them to the facilitators), the authors responded by asking the other participants to present how they would recommend that the issue be handled. In this respect, there is a clear priority of the process and quality of participation over specific details of the design outcome. The end result, however, was greater buy-in, more compliance, and improved outcomes for their hospitals. Like the PD case study, this process took significantly longer and, arguably, represented a more realistic process for changing long-standing ways of working (see also Carlgren [52]). The authors note that other teams using DT in their work at Kaiser Permanente had experienced similar disengagement, where the innovations lacked sustainability in sites outside the origin of development. Lin et al [51] note the need for the design to arise out of end users’ own concerns, which arguably is the central tenant of DT.

While the Das and Svanaes PD project [53] involved a limited number of end users, there was transparency in the origin of design ideas. The DT and PD teams began with similar processes (eg, interviews, observations) but then diverged, with the PD researchers working with end users in idea generation whereas the DT team did this internally. We are unable, however, to determine whether the more participatory process employed by Das and Svanaes resulted in greater uptake and buy-in by end users with the final implementation; as with much research in PD, the focus of the paper is on how the methods of participation they used elicited valuable insights for design rather than the success of the resulting system in use.

## Discussion

The Lin et al [51] case study highlights that DT approaches can be employed in ways that limit the participation of non-designers to expert informants of the contexts of use, or evaluators of ideas, that have been generated through the process. This traditional, less participatory application of DT appears more likely to encounter difficulties and/or resistance in a health care context. The case study contains clear lessons for design of e-mental health and well-being interventions, many of which will be implemented in organizational contexts. Design solutions not generated with end users themselves are more likely to fail, a notion that receives support elsewhere in the literature [38,54]. The manner and method in which design ideas are introduced, discussed, and progressed requires careful consideration for technology design in mental health, a context that is principally composed of highly educated and experienced health professionals who are afforded considerable autonomy in their daily work. Modern application of PD in health intervention research leverages professional and consumer expertise to collaboratively achieve good design outcomes. Its egalitarian mind-set and process may be better suited to mental health professionals who regularly rely on their clinical judgement and expertise in high risk, complex situations. Drawing from and appreciating this experience through meaningful collaboration, as demonstrated in the Das and Svanaes [53] PD project and the more inclusive process of the NKEplus redesign, is likely to yield greater uptake and longevity of research outputs in context. This claim is supported by Lin et al who, along with other DT experts in their organization, report experiencing ongoing difficulties with bedding down change initiatives that result from traditional expert-led application of DT methods.

One may ask, in promotion and practice of traditional DT methods, are we unhelpfully replacing one expert-led model in health research with another? The difficulty experienced by the DT teams throughout Kaiser Permanente highlight potential inherent limitations in the DT methods for a health care context and the level of experience required for effective practice (or adaptation) of them. The highly experienced team that led this project reported many problems with generating long-term change as a result of the innovation that came out of their DT cycle(s). Furthermore, in selecting the case study for this paper, DT projects in a health care context were scarce and novice-led DT projects were nonexistent. In light of these findings, the claims of novice user uptake of DT seem optimistic at best.

The Das and Svanaes [53] project demonstrates the value of PD for buy-in and uptake of interventions; however, the traditional focus on process over outcome in PD research leaves unresolved questions around its utility as a methodology for intervention design, development, and implementation. From a non-design specialist perspective, the Das and Svanaes paper [51] clearly articulated their methods and techniques, however, the method cards in the DT toolkits more clearly articulate the designer skill set (ie, the tacit mind-sets and capabilities or what to look for and why). For example, the d.School Bootcamp Bootleg [47] articulates mind-sets and behaviors, particularly around empathy and quick-and-dirty prototyping (and show don’t tell), which may combine nicely with the participatory, egalitarian elements of PD. In the absence of these designerly mind-sets, it is likely that the early interview and observation work could miss the design perspective and end up an ethnographic study. This is problematic as, while this phase of the design cycle possesses an ethnographic-like quality in that it attempts to better understand existing workflows, circumstances, and people’s subjective experience, it should also elicit data around tensions, contradictions, and opportunities for design—crucial design elements that may be overlooked with a purely ethnographic mind-set.

### Conclusions

The very clear articulation of mind-set (and output expected from a particular method/technique) in the DT toolkits (such as the progression from empathize to point-of-view to ideate in the early stages of a DT project) provide clarity and design direction for the ethnographic and observational components of design projects. Much can be learned from this approach in health intervention design research and the value of ongoing dialogue and collaboration between health and design research disciplines in this space should not be underestimated. As discussed in the introduction, however, access to mental health workplaces for observation is not an easily negotiated proposition. In comparison to DT, the more integrated nature, and egalitarian purpose, of PD projects supports greater opportunities for meaningful collaboration between research and clinical practice. If the mental health workforce can see the value of the project (because they have played key roles in its origin), research projects stand a greater chance of accessing the individuals and environments they require for intervention design.

We might also note in conclusion that there is a sentiment within the design research community that the notion of design thinking is in danger of being superficially reduced to a toolbox of easy-to-apply methods that appear to offer recipe-like solutions to a vast range of complex problems. This is a serious concern, and it is worth pointing out that the curricula of most studio-based design programs in higher education neither contain nor resemble what has become visible as design thinking. The existence of resources like Stanford’s d.School Bootcamp Bootleg, a suite of methods that are freely distributed and packaged in step-by-step instructions is, we believe, a generous gift to the community at large. But their value in application to new and complex spaces (mental health services being our foremost concern in this paper) must be tied to the mind-set in which they are employed. In this domain, such a mind-set ought to draw from both studio-based design disciplines that have given rise to design thinking and from the social and ethical imperatives of participatory design. From design thinking disciplines, such a mind-set incorporates an appreciation of the nature of design as an exploratory, iterative, uncertain, and social form of inquiry (and synthesis) that is never perfect and never quite finished. This understanding of design practice is articulated well in Schön [55]. From participatory design disciplines, the mind-set involves an appreciation that good design emerges from thoughtful and humble facilitation, that participants need to be given the opportunity to take multiple and active roles in all aspects of design, and that shared ownership over proposals for change can be a more valuable form of innovation than technological novelty and disruption. If the design object and/or outcomes require widespread organizational uptake, handing over control of the design process (as in PD) in appreciation of this context can be just as important as the eventual product in generating (and managing) the change.

We in the e-mental health research community must debate and reflect on exactly what we are trying to achieve through the adoption of DT or PD in our work. Do we seek to incorporate new and potentially disruptive ways of working because they are freely available and promise (narrowly defined ideas of) innovation? Or are we in pursuit of methods and interventions that privilege the needs, voice, and contribution of health consumers and professionals? Moreover, from an ethical and moral perspective, egalitarian ways of working such as those exemplified by PD also represent a promising opportunity to redress the legacy of consumer disempowerment in mental health.

## Abbreviations

CRC: Cooperative Research Centre
DT: design thinking
NKE: Nurse Knowledge Exchange
PD: participatory design
